# Supplementary figures and images for: Optical tissue clearing and 3D imaging of intact primate testicular tissue: a novel technology development
Source: PLoS One. 2025 Dec 8;20(12):e0327287. doi: 10.1371/journal.pone.0327287 (PMC12685185; doi:10.1371/journal.pone.0327287)

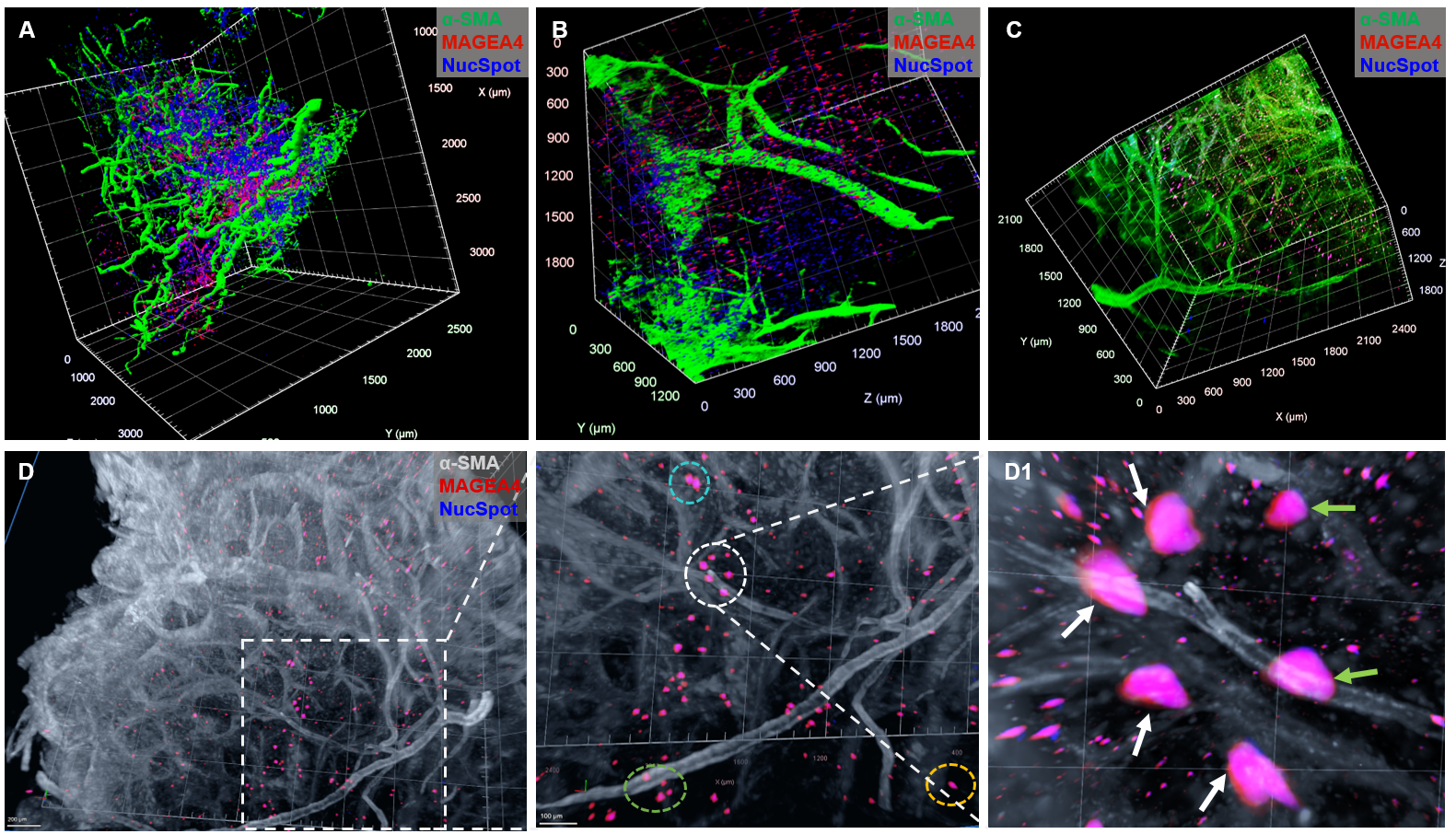

Supplement: S1 Fig — Vascular network (green) in close association with MAGEA4-positive spermatogonia (red) and other testicular cells (blue) in volumetric projections in (A) human transgender model, tissue dimensions: 3 mm x 3 mm x 4 mm {width (X) x height (Y) x depth (Z)} totaling 36 mm3, (B) marmoset, tissue dimensions: 3.2 mm x 3.2 mm x 2.8 mm {width (X) x height (Y) x depth (Z)} totaling 29 mm3, and (C) macaque, tissue dimensions: 3.2 mm x 3.2 mm x 2.4 mm {width (X) x height (Y) x depth (Z)} totaling 25 mm3. The cells that appear magenta in color are spermatogonia that are stained positive for both MAGEA4 and nucleardye. Scale bars: 50 µm. (D) Macaque ITT showing distribution of testicular cells. Clones of MAGEA4-positive spermatogonia were observed occurring as single cells, orange circle; in pairs, cyan circle; or in groups of four, green circle or eight cells (not shown) as marked by the circles. (D1) Inset of a group of six spermatogonia, notice that spermatogonia marked with green arrows have been sliced during collection of the tissue. Scale bars: 200 and 100 µm (inset). (TIF) [file pone.0327287.s005.tif]

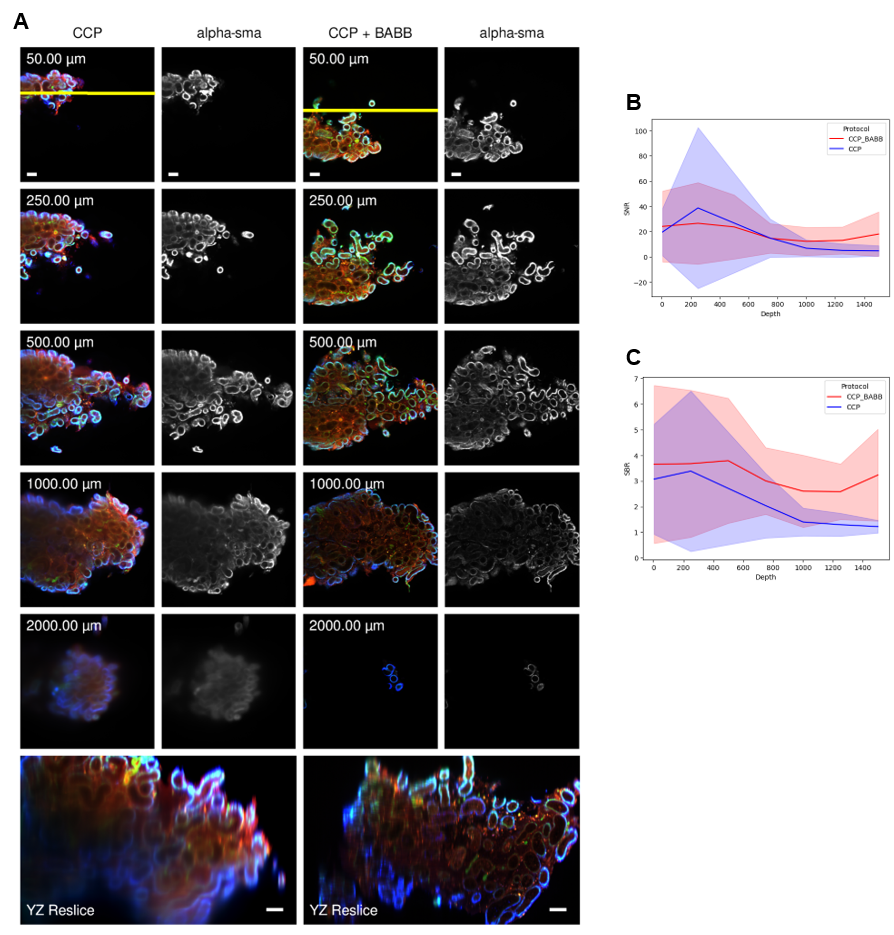

Supplement: S2 Fig — Comparison of commercial clearing kit alone (CCP) with commercial clearing kit followed by BABB clearing (CCP + BABB) in human testes. (A) Representative optical sections at 50, 250, 500, 1000, and 2000 µm depth showing α-SMA–Cy3 (green), vimentin–488 (red), and MAGEA4–AF647 (far-blue) channels for both clearing conditions. Grayscale insets display the α-SMA channel alone. YZ reslice along the yellow line in the overview image illustrates α-SMA–positive structures across tissue depth. Samples were illuminated with a single light sheet directed from the right. Scale bar, 200 µm. (B-C) Signal-to-noise (SNR) (B) and contrast (signal-to-background (SBR)) (C) measurements along the z-axis. Mean fluorescence intensity and standard deviation was quantified by selecting SMA–Cy3–positive regions outlining testicular tubules at each depth (0–1500 µm, 250 µm steps). Square ROIs (10 × 10 pixels) were measured for signal, and corresponding background ROIs were obtained by translation into the tubule interior (unstained region). Shown are SNR and SBR for 20 α-SMA–Cy3–positive regions and corresponding background regions for each z-slice + /- SD. (TIF) [file pone.0327287.s006.tif]

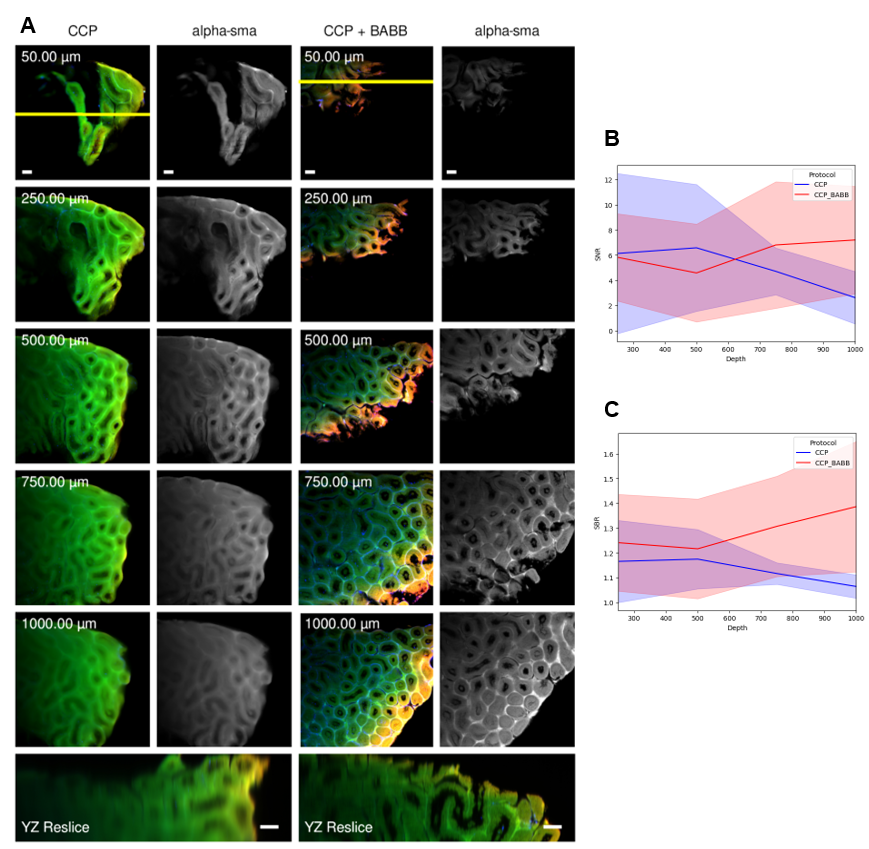

Supplement: S3 Fig — Comparison of commercial kit clearing alone (CCP) with commercial kit clearing followed by BABB clearing (CCP + BABB) in marmoset testes. (A) Representative optical sections at 50, 250, 500, 1000, and 2000 µm depth showing α-SMA–Cy3 (green), vimentin–488 (red), and MAGEA4–AF647 (far-blue) channels for both clearing conditions. Grayscale insets display the α-SMA channel alone. YZ reslice along the yellow line in the overview image illustrates α-SMA–positive structures across tissue depth. Samples were illuminated with a single light sheet directed from the right. Scale bar, 200 µm. (B-C) Signal-to-noise (SNR) (B) and contrast (signal-to-background (SBR)) (C) measurements along the z-axis. Mean fluorescence intensity and standard deviation was quantified by selecting SMA–Cy3–positive regions outlining testicular tubules at each depth (250–1000 µm, 250 µm steps). Square ROIs (10 x 10 pixels) were measured for signal, and corresponding background ROIs were obtained by translation into the tubule interior (unstained region). Shown are SNR and SBR for 20 α-SMA–Cy3–positive regions and corresponding background regions for each z-slice + /- SD. (TIF) [file pone.0327287.s007.tif]

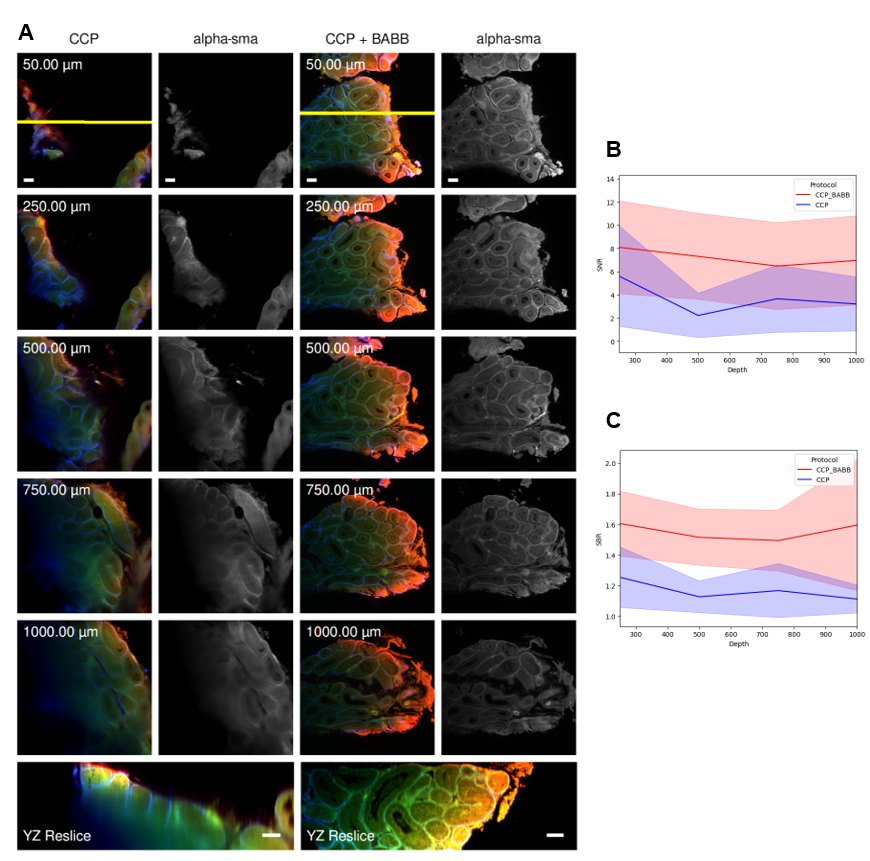

Supplement: S4 Fig — Comparison of commercial kit clearing alone (CCP) versus commercial kit clearing followed by BABB clearing (CCP + BABB) in macaque testes. (A) Representative optical sections at 50, 250, 500, 1000, and 2000 µm depth showing α-SMA–Cy3 (green), vimentin–488 (red), and MAGEA4–AF647 (far-blue) channels for both clearing conditions. Grayscale insets display the α-SMA channel alone. YZ reslice along the yellow line in the overview image illustrates α-SMA–positive structures across tissue depth. Samples were illuminated with a single light sheet directed from the right. Scale bar, 200 µm. (B-C) Signal-to-noise (SNR) (B) and contrast (signal-to-background (SBR)) (C) measurements along the z-axis. Mean fluorescence intensity and standard deviation was quantified by selecting SMA–Cy3–positive regions outlining testicular tubules at each depth (250–1000 µm, 250 µm steps). Square ROIs (10 × 10 pixels) were measured for signal, and corresponding background ROIs were obtained by translation into the tubule interior (unstained region). Shown are SNR and SBR for 20 α-SMA–Cy3–positive regions and corresponding background regions for each z-slice + /- SD. (TIF) [file pone.0327287.s008.tif]

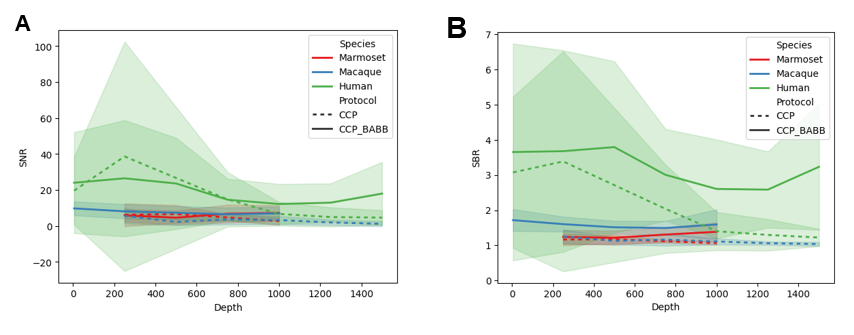

Supplement: S5 Fig — (A) Signal-to-noise (SNR) and (B) and contrast (signal-to-background (SBR). (TIF) [file pone.0327287.s009.tif]

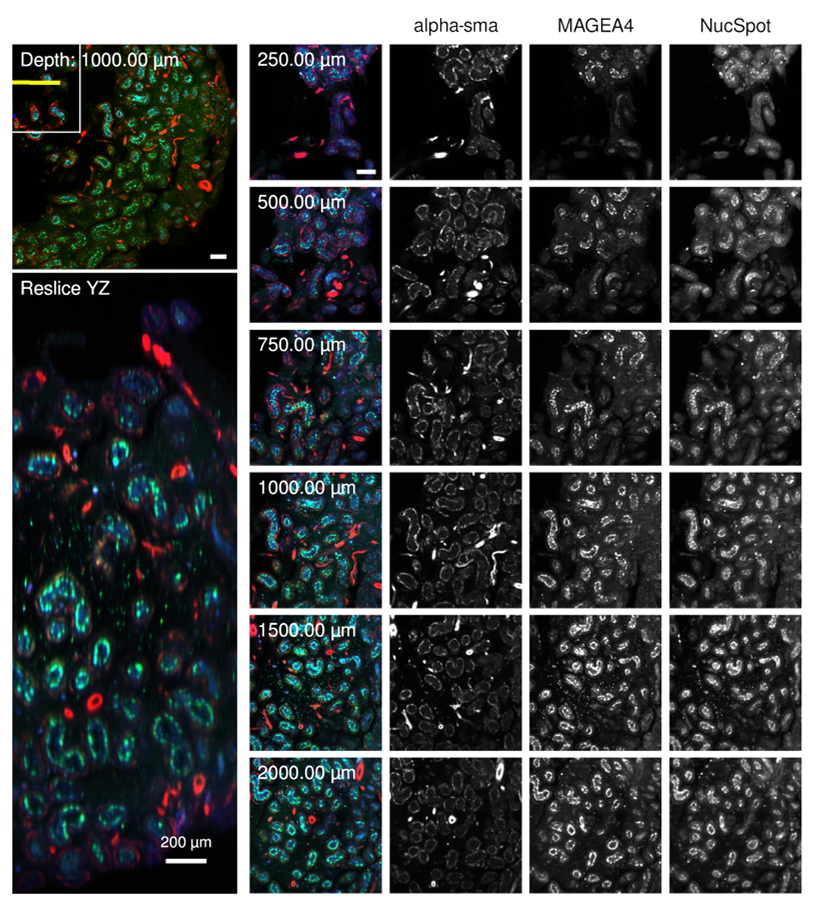

Supplement: S6 Fig — Overview image at 1000 µm depth (upper left) with zoomed regions (white rectangle) shown at different depths across the stack (right panels) for human testes. All three fluorescence channels are displayed in color (α-SMA, red; MAGEA4, green; NucSpot, blue) and individually in grayscale, with brightness and contrast kept consistent across depths. A YZ reslice along the yellow line in the overview (lower left) illustrates signal distribution through the tissue. Scale bar, 200 µm. (TIF) [file pone.0327287.s010.tif]

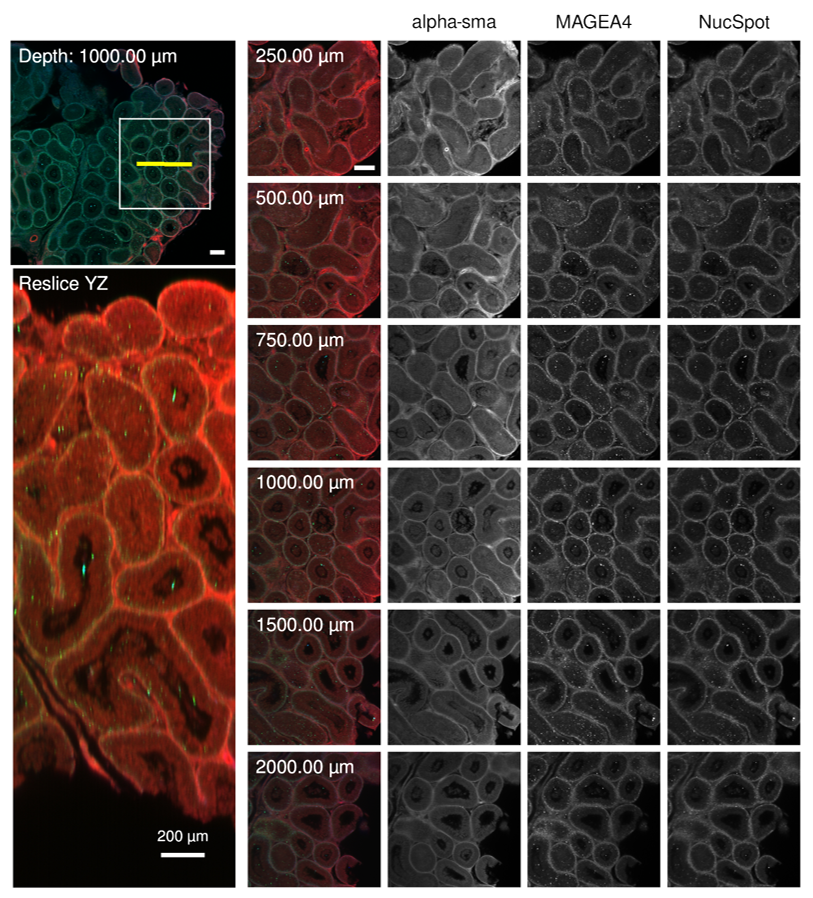

Supplement: S7 Fig — Overview image at 1000 µm depth (upper left) with zoomed regions (white rectangle) shown at different depths across the stack (right panels) for marmoset testes. All three fluorescence channels are displayed in color (α-SMA, red; MAGEA4, green; NucSpot, blue) and individually in grayscale, with brightness and contrast kept consistent across depths. A YZ reslice along the yellow line in the overview (lower left) illustrates signal distribution through the tissue. Scale bar, 200 µm. (TIF) [file pone.0327287.s011.tif]

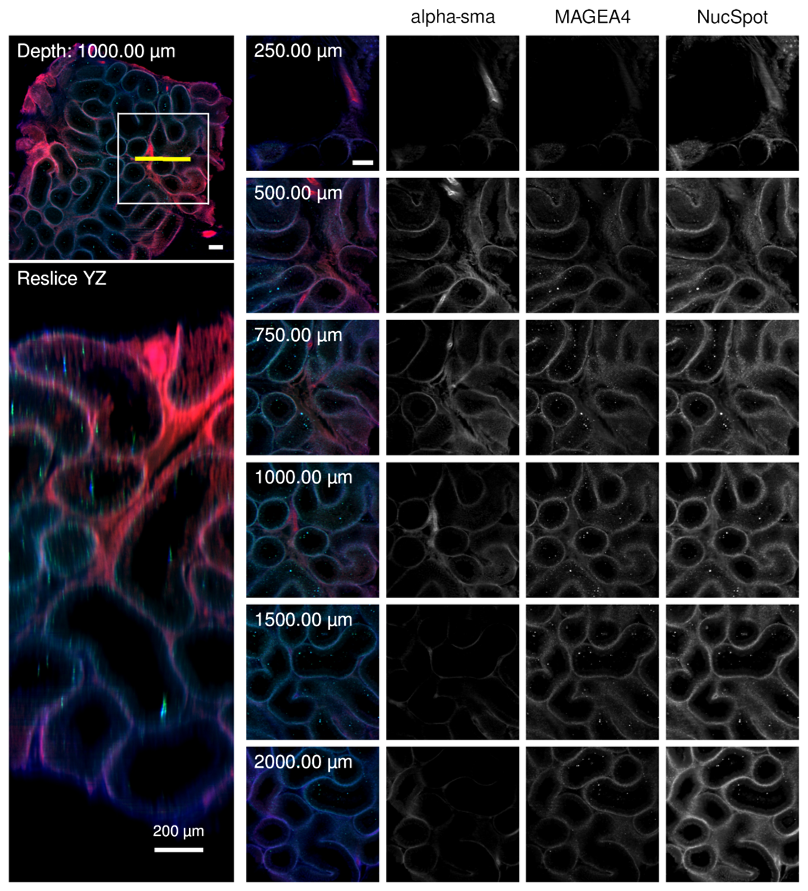

Supplement: S8 Fig — Overview image at 1000 µm depth (upper left) with zoomed regions (white rectangle) shown at different depths across the stack (right panels) for macaque testes. All three fluorescence channels are displayed in color (α-SMA, red; MAGEA4, green; NucSpot, blue) and individually in grayscale, with brightness and contrast kept consistent across depths. A YZ reslice along the yellow line in the overview (lower left) illustrates signal distribution through the tissue. Scale bar, 200 µm. (TIF) [file pone.0327287.s012.tif]

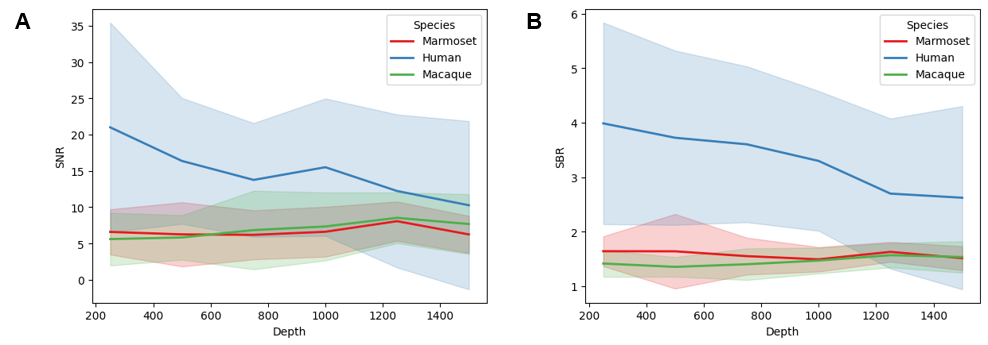

Supplement: S9 Fig — Signal-to-noise (SNR) (A) and contrast (signal-to-background (SBR)) (B) measurements along the z-axis. Mean fluorescence intensity and standard deviation was quantified by selecting SMA–Cy3–positive regions outlining testicular tubules at each depth (250–1500 µm, 250 µm steps). Square ROIs (10 × 10 pixels) were measured for signal, and corresponding background ROIs were obtained by translation into the tubule interior (unstained region). Shown are SNR and SBR for 20 α-SMA–Cy3–positive regions and corresponding background regions for each z-slice + /- SD. (TIF) [file pone.0327287.s013.tif]
