# Supplementary material for: Optical tissue clearing and 3D imaging of intact primate testicular tissue: a novel technology development
Source: PLoS One. 2025 Dec 8;20(12):e0327287. doi: 10.1371/journal.pone.0327287 (PMC12685185; doi:10.1371/journal.pone.0327287)
Supplement: S1 Table — (DOCX) [file pone.0327287.s014.docx]

| **No.** | **Antibody name** | **Catalogue number** | **Company** | **Dilution** |
| --- | --- | --- | --- | --- |
|  | **Germ cell markers** | | | |
| 1 | Mouse-monoclonal -anti-MAGEA4 | Gift from Prof. G.C. Spagnoli, Switzerland | | 1:20 |
| 2 | Polyclonal Rabbit anti‑Human HIWI2 / PIWIL4 | LS‑C482396-100 | LS Bio | 1:100 |
|  | **Somatic cell markers** | | | |
| 3 | Anti-vimentin VHH/ Nanobody -Label Atto488 | vba488 | Proteintech | 1:200 |
| 4 | Rabbit Polyclonal Antibody anti-SOX9 | AB5535 | Merck | 1:200 |
| 5 | Mouse-monoclonal α-SMA-Cy3™ | C6198 | Sigma-Aldrich | 1:200 |
| 6 | Mouse-anti-BrdU | B2531 | Sigma Aldrich | 1:50 |
|  | **Control and secondary antibodies** | | | |
| 7 | Mouse IgG Control Antibody, Unconjugated | Sigma-Aldrich | I5381 |  |
| 8 | Rabbit IgG Control Antibody, Unconjugated | Sigma-Aldrich | I5006 |  |
| 9 | Donkey α-mouse Alexa Fluor 488 | 715546150 | Jackson ImmunoResearch | 1:100 |
| 10 | Donkey α-rabbit Alexa Fluor 647 | A31573 | Invitrogen | 1:100 |
|  | **Other reagents** | | | |
| 11 | VECTASHIELD® Antifade Mounting Medium with DAPI | VEC-H-1200 | Vector Laboratories |  |
| 12 | Vectashield mounting medium WITHOUT DAPI | VEC-H-1000 | Vector Laboratories |  |
| 13 | Normal Donkey Serum | 017-000-121 | Jackson ImmunoResearch |  |
