# Supplementary material for: Optical tissue clearing and 3D imaging of intact primate testicular tissue: a novel technology development
Source: PLoS One. 2025 Dec 8;20(12):e0327287. doi: 10.1371/journal.pone.0327287 (PMC12685185; doi:10.1371/journal.pone.0327287)
Supplement: Attachment — Submitted filename: Response Letter Revision 2 PlosOne_05_11_2025.docx [file pone.0327287.s017.docx]

To Su-Ren Chen
Academic Editor PLOS ONE

**RE: Response Letter**

PONE-D-25-22183

Title: Optical tissue clearing and 3D imaging of intact primate testicular tissue: a novel technology development

Dear Mrs. Chen.

We appreciate the responses of both reviewers and are delighted that reviewer 1 accepted our revision. We have dealt with the comments of reviewer 2 during a second round of revision and resubmit the revised manuscript. You find our response to the three comments and appropriate changes in the text.

**Response to Reviewers**

Reviewer #1: The authors have adequately addressed all the comments; therefore, no further revisions are needed on my part

Reviewer #2: Thank you to the authors for thoughtfully addressing each of my previous comments. I have a couple of additional recommendations:

Comments by reviewer 2

1) Lines 466–482: The purpose of this paragraph is to compare PT-CLEAR3D with the other clearing techniques and to justify why PT-CLEAR3D represents the optimal approach. However, with the inclusion of new information regarding the analysis of the different clearing techniques, as well as the clarification that the testing was conducted only on testis tissue and not on other tissue types, the concluding sentence of the paragraph now appears somewhat disjointed. I recommend revising the structure of this final paragraph to improve its coherence and logical flow.

**Answer:** We have rewritten the summarizing paragraph to increase the coherence (Lines 455-476).

2) I commend the authors for their additions in response to the first reviewer, which in my view clearly demonstrate the benefit of additional BAAB clearing following the use of the commercial clearing kit in primate testis tissue (Figs. S2–S5). However, it is unexpected that PT-CLEAR3D was omitted from this analysis. Including PT-CLEAR3D in these comparative analyses would substantially strengthen the overall message of the manuscript.

**Answer:** We appreciate the reviewer’s commendation for demonstrating BABB’s beneficial effect on rescuing clearing efficiency following the use of the commercial kit (Figs. S2–S5).

We are showing the results of our PT-CLEAR3D in Fig. S6-S9. The additional figures S2-S9 were added on request by the first reviewer to address signal to background ratio and depth. This has determined the order of the figures added during revision 1.

To address the reviewer’s suggestion, we have now expanded the manuscript to provide detailed descriptions of the comparative analyses in the main text (Lines 443-454), which are already visually represented in figures illustrating depth-dependent image quality (Figs. S2–S9). We assessed signal-to-noise ratio (SNR) and signal-to-background ratio (SBR) across all clearing conditions – CCP, CCP + BABB, and PT-CLEAR3D – in corresponding figures. While we elected not to aggregate all measurements into a single figure due to substantial differences in imaging conditions (such as laser power, one-sided light sheets vs two-sided, time of acquisition) between Figs. S2–S5 and Figs. S6–S9, we have revised the manuscript to detail results for all conditions. We acknowledge that this introduces potential limitations in direct SNR/SBR comparison due to variations in experimental parameters. Nevertheless, we believe the reported trends – including improved SNR/SBR in human tissues accompanied by slight depth-dependent decreases, and relatively lower SNR/SBR in macaque and marmoset tissues potentially attributable to less efficient antibody labelling without significant depth-dependent degradation – remain consistent across all conditions.

3) There appears to be a large standard deviation in Figs. S5 and S9. The authors should provide statistical evidence to indicate whether there are significant differences between the clearing techniques at depth.

**Answer**: We thank the reviewer for this observation. The large standard deviations in Figures S5 and S9 reflect the intrinsic variability of the measurements rather than replicate-based variation. These measurements were performed on representative samples from both clearing protocols.

Signal intensity was quantified at 20 randomly selected positions per optical plane at different depths throughout the tissue. However, testicular tissue is inherently heterogeneous, and local variations in cell density and refractive index can influence light propagation. Given the effective tissue thickness shown, each measurement point may differ, leading to variable fluorescence attenuation. Additionally, regions closer to the periphery of the light sheet generally receive stronger illumination. At the same time, attenuation occurs not only along the z-axis (depth) but also laterally (y-axis) due to imperfect clearing.

Consequently, a statistical comparison between clearing methods based on single-sample datasets does not provide a meaningful or reliable assessment of performance. Instead, qualitative evaluation of the raw LSFM images (Fig. S2-S4, S6-S8) more clearly illustrates the differences in optical transparency and image quality between the protocols—particularly the presence of blur and reduced resolution in less efficiently cleared samples, which may not be fully captured by simple intensity-based metrics.
